# Insight into the Prevalence of Extended-Spectrum β-Lactamase-Producing Enterobacteriaceae in Vegetables: A Systematic Review and Meta-Analysis

**DOI:** 10.3390/foods13233961

**Published:** 2024-12-08

**Authors:** Sebolelo Jane Nkhebenyane, Ntelekwane George Khasapane, Kgaugelo Edward Lekota, Oriel Thekisoe, Tsepo Ramatla

**Affiliations:** 1 Centre for Applied Food Safety and Biotechnology, Department of Life Sciences, Central University of Technology, 1 Park Road, Bloemfontein 9300, South Africa; snkheben@cut.ac.za (S.J.N.); nkhasapane@cut.ac.za (N.G.K.); 2 Unit for Environmental Sciences and Management, North-West University, Potchefstroom 2531, South Africa; lekota.lekota@nwu.ac.za (K.E.L.); oriel.thekisoe@nwu.ac.za (O.T.)

**Keywords:** global prevalence, vegetables, enterobacteriaceae, extended-spectrum β-lactamase

## Abstract

The occurrence of extended-spectrum β-lactamase (ESBL)-producing Enterobacteriaceae in vegetables is an escalating global problem. This study aimed to document the global prevalence of ESBL-producing Enterobacteriaceae in vegetables using a comprehensive meta-analysis. A web-based search of electronic databases such as ScienceDirect, Google Scholar, and PubMed was conducted using studies published between 2014 and 2024. The Preferred Reporting Items for Systematic Reviews and Meta-Analysis (PRISMA) guidelines were followed for the systematic review and meta-analysis. The Comprehensive Meta-Analysis (CMA) Ver 4.0 software was used to analyse the data. The pooled prevalence estimate (PPE) with a 95% confidence interval (CI) was calculated using the random effects model. After reviewing 1802 articles, 63 studies were carefully analyzed and were part of the comprehensive meta-analysis. The overall PPE of ESBL-producing Enterobacteriaceae (ESBL-E) was 11.9% (95% CI: 0.091–0.155), with high heterogeneity (I^2^ = 96.8%, *p* < 0.001) from 2762 isolates. The *bla*_SHV_ ESBL-encoding gene was the most prevalent, showing a PPE of 42.8% (95% CI: 0.269–0.603), while the PPE of *bla*_ampC_-beta-lactamase-producing Enterobacteriaceae was 4.3% (95% CI: 0.025–0.71). Spain had a high ESBL-E PPE of 28.4% (0.284; 95% CI: 0.057–0.723, I^2^ = 98.2%), while China had the lowest PPE at 6.4% (0.064; 95% CI: 0.013–0.259, I^2^ = 95.6%). Continentally, the PPE of ESBL-E was significantly higher in reports from South America at 19.4% (95% CI: 0.043–0.560). This meta-analysis showed that ESBL-E in vegetables increased by 9.0%, 9.8%, and 15.9% in 2018–2019, 2020–2021, and 2022–2024, respectively. The findings emphasize the potential risks of consuming raw or inadequately cleaned produce and the importance of vegetables as ESBL-E reservoirs. Our work calls for immediate attention to food safety procedures and more thorough surveillance as antibiotic resistance rises to reduce antimicrobial resistance risks in food systems.

## 1. Introduction

The Enterobacteriaceae family of Gram-negative bacteria produces enzymes called extended-spectrum beta-lactamases (ESBLs), which provide the bacteria with strong resistance to various antibiotics. The Enterobacteriaceae family, which carries ESBL genes in their plasmids or chromosomes, produces β-lactam-hydrolyzing enzymes, and they are classified as high-priority pathogens by the World Health Organization (WHO) [1]. They differ from each other in terms of sequence homology, inhibitor profiles, and substrate profiles [2]. Moreover, they are a class of enzymes that produce resistance to monobactams and oxyimino-cephalosporins but not to cephamycins or carbapenems [3]. The complexity of treating infections brought on by these bacteria is further increased because plasmids containing ESBL genes frequently contain other genes that confer resistance to different types of antibiotics [4]. Ambler class A ESBLs (ESBLA), miscellaneous ESBLs (ESBLM), and ESBLs that degrade carbapenems (ESBLCARBA) are the three main categories into which ESBLs have recently been divided [5]. Temoniera (TEM), cefotaxime-M (CTX-M), and various sulfhydryl reagent variable (SHV) β-lactamases belong to the ESBLA group, which accounts for the majority of ESBLs worldwide [1,3].

The emergence of ESBL-producing Enterobacteriaceae (ESBL-E) has driven a global health crisis, leading to higher morbidity rates, mortality, and antibiotic treatment failures [1]. Five major nosocomial pathogens produce ESBLs, including *Acinetobacter baumannii*, *Escherichia coli*, *Klebsiella pneumoniae*, *Pseudomonas aeruginosa*, and *Enterobacter* spp. [6]. These pathogens are responsible for numerous infection outbreaks worldwide and challenge infection control [7]. Alarmingly, recent studies suggest that antibiotic-resistant bacteria (ARBs) and antibiotics (ARGs) are increasingly present in vegetables, posing a serious health risk since vegetables are often eaten raw or minimally processed [8,9]. Furthermore, it has been suggested that environmental and soil samples represent important reservoirs for ARBs and ARGs [8,10].

Several methods for detecting ESBL-E have been used by previous studies, such as tests recommended by CLSI for ESBL screening, including Kirby–Bauer disks and Vitek system [1,10], the double-disk synergy test (DDST), the double-disk synergy method, or E-test ESBL strips [4,7,8]. Polymerase chain reactions with restriction fragment length polymorphism analysis, PCR with single-strand conformation polymorphism analysis, oligotyping, DNA probes, and real-time PCR are other techniques that can be used [1,10]. ESBL has also been successfully detected using matrix-assisted laser desorption ionization–time of flight mass spectrometry (MALDI-TOF) [11]. Notably, the European Committee on Antimicrobial Susceptibility Test (EUCAST) has established guidelines for ESBL detection [1].

Several systematic review and meta-analysis studies have been conducted on ESBL-E in Africa’s water–plant–food interface [12] and wastewater [13] in community-based and clinical trials [14]. However, there is no information on comprehensive data to estimate the global prevalence of ESBL-E in vegetables. Thus, this current systematic review and meta-analysis sought to provide a comprehensive overview of the global prevalence of ESBL-E in vegetables.

## 2. Materials and Methods

### 2.1. Study Design and Systematic Review Protocol

Using PRISMA (Preferred Reporting Items for Systematic Reviews and Meta-Analyses) guidelines [15], the data extraction, review, and analysis included searching database systems for potentially relevant articles, assessing their relevance, and assessing whether they were relevant to the review, which was confirmed using a checklist (Supplementary Table S1).

### 2.2. Search Strategy

This study utilized multiple databases: Google Scholar, PubMed, and ScienceDirect. Searches of the literature were conducted using keywords comprising “Extended-spectrum β-lactamase” OR ESBL AND Enterobacteriaceae, “Extended-spectrum β-lactamase” OR ESBL-E AND Extended spectrum β-lactamase” OR ESBL AND Enterobacteriaceae AND vegetables, “Beta-lactamases” OR “β-lactamases” AND Enterobacteriaceae AND vegetables. The last search took place on 22 August 2024.

### 2.3. Study Selection

The three authors, TR, GK, and OT, checked the suitability of journal article titles and abstracts for their suitability for the inclusion and exclusion criteria. All studies from the search were independently analyzed by title, abstract, and selected full text by two reviewers, and a third reviewer adjudicated any discrepancies. Full-text journal articles published in English were included, while reviews, conference abstracts, and chapter books were excluded. Journal articles were selected for full-text review if they were research studies on ESBL-E in vegetables.

### 2.4. Inclusion and Exclusion Criteria

The following criteria had to be met for the studies to be included: (a) studies looking into the prevalence of ESBL-E in vegetables, (b) studies published as a full text, (c) studies conducted from 2014, (d) studies conducted on vegetables, and (e) studies with the total number of isolates. The exclusion criteria were as follows: (f) studies with no total number of isolates, (g) studies conducted in humans and animals, and (h) book chapters, experiments, reviews, and case reports were excluded.

### 2.5. Data Extraction 

Two independent authors (JN and TR) extracted and summarized the following data from the final selected studies. (a) author names, (b) year of publication, (c) location, (d) type of samples, (e) year of publication, (f) ESBL identification methods, (g) positive samples of ESBL-E in vegetables, (h) total number of ESBL-E in vegetables, and (i) antibiotic resistance genes, which are encoded for ESBL resistance. Microsoft Excel was used to compile the recorded data for additional analysis. 

### 2.6. Outcome Measurements

The primary findings of this study were the prevalence of ESBL-E in vegetables. ESBL resistance genes and multidrug resistance (MDR) were studied from 1 January 2014 to 22 August 2024.

### 2.7. Quality Assessment of Included Studies

The bias risk was evaluated in each study using a critical appraisal tool created by the Joanna Briggs Institute (JBI) [16]. Quality assessment was conducted using the JBI critical appraisal checklist for cross-sectional studies using nine criteria. This tool has question types of “Yes”, “No”, “Unclear”, or “Not applicable”, and the score is given as 1 for “Yes” and 0 for “No”. Studies were classified as low-, medium-, and high-quality if the summed scores were 0–4, 5–7, and 7–9, respectively. Articles with high and medium risks of bias were excluded from the study, and only articles with a low risk of bias were included.

### 2.8. Data Synthesis and Data Analysis

A meta-analysis of the prevalence of ESBL-E in vegetables was conducted using the Comprehensive Meta-Analysis software v.4.0 (https://www.meta-analysis.com/) [17,18]. The pooled prevalence of ESBL-E isolates was measured, and subgroup analysis was performed by country, continent, ESBL-resistant genes, and samples collected. Random effects models created forest plots showing the study-specific effect sizes with a 95% confidence interval (CI) for the pooled prevalence estimate (PPE). The I^2^ statistic was used to measure heterogeneity between studies. A value of 0% indicates no heterogeneity, while values of 25%, 50%, and 75% [19] correspond to low, moderate, and high heterogeneity, respectively. The *p*-values correspond to the heterogeneities between studies from a chi-square test of the null hypothesis of no heterogeneity.

### 2.9. Publication Bias

The Egger regression test and the Beg and Mazumdar rank correlation test were used to assess publication bias further and check for symmetry using funnel plots [20]. A *p*-value ≥ 0.05 indicated a lack of publication bias. Plotting effect sizes against their precision or the reciprocal of standard errors is a common use for funnel plots. By constructing a funnel plot with the observed studies and the imputed studies required to establish the absence of bias, we were able to produce the best estimate of the unbiased pooled effect size.

## 3. Results

### 3.1. Search and Screen Outcomes

**Figure 1:**
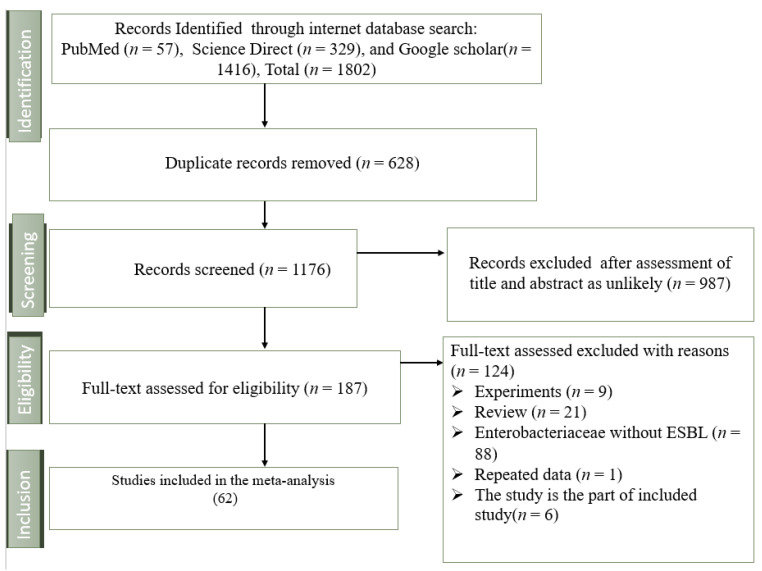
A PRISMA flowchart illustrating the study selection process.

A total of 1802 articles were initially retrieved from the database searches, with 987 duplicates subsequently removed. After full-text screening, 124 were excluded for various reasons, leaving 63 studies eligible for data extraction and analysis (Figure 1).

### 3.2. Characteristics of Eligible Studies

**Figure 2:**
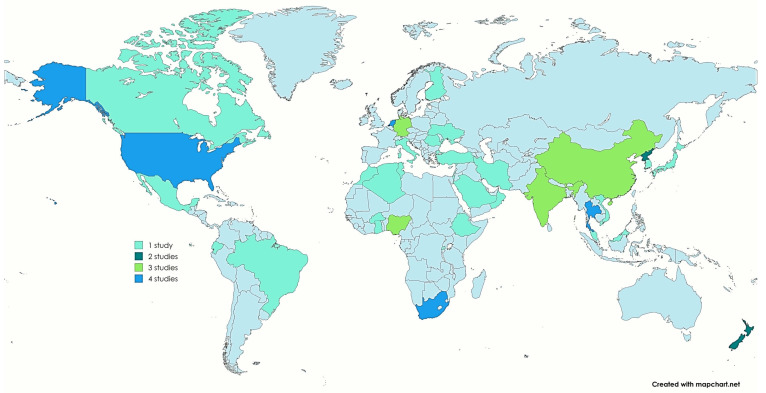
This global map displays the number of studies from various countries documenting the prevalence of ESBL-E in vegetables. (https://www.mapchart.net/world.html (accessed on 27 October 2024).

The number of ESBL-E isolates per study ranged from 1 to 1224, with all studies published between 2014 and 2024. The characteristics of the studies included in this study are presented in Table 1. A total of 63 studies were conducted across different continents, including Africa (*n* = 14), Asia (*n* = 23), Europe (*n* = 16), North America (*n* = 5), and South America (*n* = 4), with one study spanning Asia and Switzerland (Figure 2). The eligible studies predominantly report on the prevalence of ESBL-E isolates in vegetables such as carrots, lettuce, radish, spring onions, cabbage, cucumbers, cornichons, lettuce, spinach, parsley, peppers, tomatoes, alfalfa sprout, broccoli sprout, radish sprout, rape sprout, and red kohlrabi sprout, etc., across different regions worldwide. Only studies published in English that focused on antibiotic resistance were included. All journal articles were published between 2014 and 2024.

### 3.3. Results of the Meta-Analysis on Overall Prevalence

**Figure 3:**
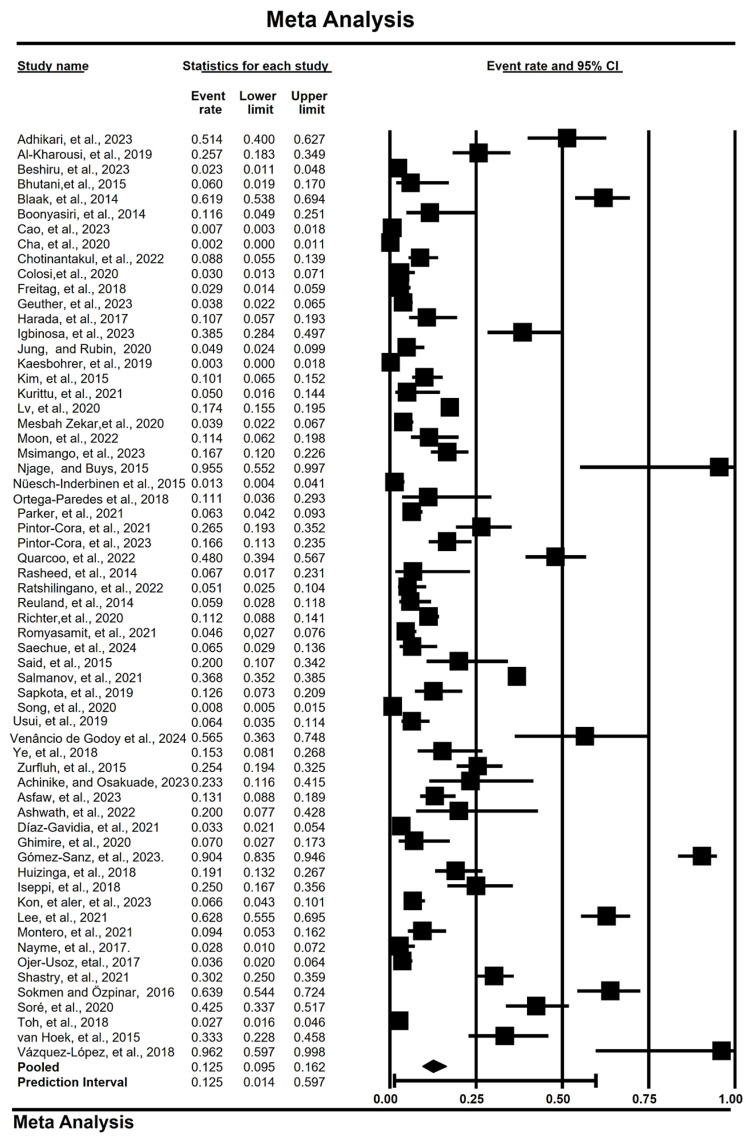
Forest plot showing the overall pooled prevalence of ESBL-producing Enterobacteriaceae. The squares demonstrate individual point estimates. The diamond at the base indicates the pooled estimates from the overall studies [21,22,23,24,25,26,27,28,29,30,31,32,33,34,35,36,37,38,39,40,41,42,43,44,45,46,47,48,49,50,51,52,53,54,55,56,57,58,59,60,61,62,63,64,65,66,67,68,69,70,71,72,73,74,75,76,77,78,79,80,81,82].

High heterogeneity was observed in the studies looking at the prevalence of extended-spectrum β-lactamase producing Enterobacteriaceae in vegetables depending on factors like overall results, methods, years, and some countries and continents (Table 2). The pooled prevalence estimate (PPE) of ESBL-E in vegetables and a summary of the subgroup analysis are presented in Table 2. A total of 2762 isolates were identified as ESBL-E from 62 studies, with a PPE of 12.5% (0.125; 95% CI: 0.095–0.162, I^2^ = 96.9%) (Table 2 and Figure 3).

### 3.4. Subgroup Analyses

#### 3.4.1. Subgroup Analysis of ESBL-Producing Enterobacteriaceae

Ten diagnostic methods were employed to identify ESBL-E (Table 2). The highest overall pooled estimate was observed in studies utilising the disk diffusion technique, with a PPE of 17.0% (0.170; 95% CI: 0.117–0.241, I^2^ = 94.8%) from 16 studies. This was followed by CHROMagar ESBL, with a PPE of 12.6% (0. 126; 95% CI: 0.640–0.231, I^2^ = 97.8%) from 13 studies, PCR, with a PPE of 21.1% (0.211; 95% CI: 0.18–0.796, I^2^ = 98.1%) from 5 studies, and the double-disk synergy test (DDST), with a PPE of 10.6% (0.106; 95% CI: 0.660–0.166, I^2^ = 93.5%) recorded from 22 studies. Due to limited data on methods such as ChromID ESBL agar (n = 2), WGS, MIC, custom-made microtitre plates, and Etest-ESBL (one study for each), data were not calculated for these techniques.

ESBL-producing Enterobacteriaceae prevalence was highest during 2022–2024, with a PPE of 15.9% (0. 159; 95% CI: 0.0.088–0.272, I^2^ = 96.3%) across 18 studies, followed by the 2014–2015 period, with a PPE of 14.2% (0.142; 95% CI: 0.067–0.277, I^2^ = 94.9%). The lowest prevalence was recorded during 2020–2021, with a PPE of 9.8% (0.098; 95% CI: 0.060–0.156, I^2^ = 98.0%) from 17 studies (Table 2).

When analysing the prevalence data in the subgroups categorized by countries, Spain had a high PPE of 28.4% (0.284; 95% CI: 0.057–0.723, I^2^ = 98.2%), whilst China had the lowest PPE at 6.4% (0.0064; 95% CI: 0.013–0.259, I^2^ = 95.6%). The prevalence data in the subgroups classified by continent showed that studies from South America recorded the highest PPE of ESBL-E at 19.4% (0.194; 95% CI: 0.043–0.560, I^2^ = 97.7%).

#### 3.4.2. ESBL-Resistant Gene Subgroup Analysis

Using a random effects model, 14 gene subsets from three or more studies were examined to determine the prevalence of ESBL-E genes. As a result, *bl*a_SHV_ had the highest PPE at 42.8% (0.428; 95% CI: 0.269–0.603, I^2^ = 73.3%), followed by *bla*_CTX-M-15_, with a PPE of 31.0% (0.310; 95% CI: 0.190–0.463, I^2^ = 72.5%), as shown in Table 3, and the mean effect sizes are shown in Figure 3. In addition, the PPE of *bla*_AmpC_ was 4.3% (0.043; 95% CI: 0.025–0.071, I^2^ = 88.9%). However, the *bla*_CMY2_, *bla*_CTX-M-9_, *bla*_CTX-M-24_, *bla*_CTX-M-25_, *bla*_CTX-M-27_, *bla*_CTX-M-38_, *bla*_CTX-M-65_, *bla*_SHV-2_, *bla*_SHV-1_, *bla*_SHV-12_, *bla*_SHV-60_, *bla*_SHV-66_, *bla*_SHV-61_, *bla*_SHV-101_, *bla*_RAHN2_, and *bla*_SHV-116_ genes were not analyzed due to insufficient reporting.

#### 3.4.3. Subgroup Analysis by ESBL-E MDR

Multidrug resistance (MDR) is defined as resistance to three or more drugs. Of 2762 detected Enterobacteriaceae isolates, only 113 ESBL-E were confirmed in six studies. The overall pooled estimate was 49.1% (0.491; 95% CI: 0.165–0.82.5, I^2^ = 94.1%).

### 3.5. Risk of Publication Bias of Included Studies

**Figure 4:**
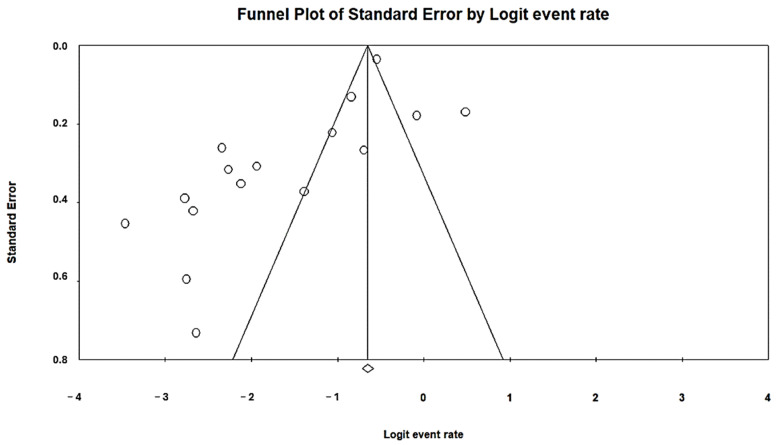
A funnel plot demonstrating the presence of publication bias in disk diffusion research.

**Figure 5:**
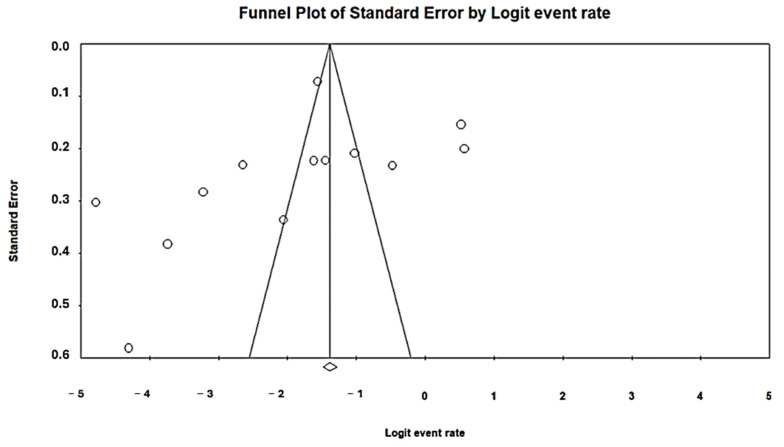
A funnel plot illustrating the studies on CHROMagar ESBL that demonstrate publication bias.

Funnel plots were used to measure publication bias and check for symmetry. The Egger regression and Begg and Mazumdar rank correlation tests supported this further. The publication bias was examined using a funnel plot. All of the included studies were distributed almost symmetrically on both sides of the funnel plot (Figure 4 and Figure 5). This review of the data published between 1 January 2014 and 22 August 2024 presents key findings on ESBL-E in vegetables, suggesting a relatively low potential for publication bias (*p*-value < 0.021) for both the disk diffusion and CHROMagar ESBL methods.

## 4. Discussion

This systematic review of data published between 1 January 2014 and 22 August 2024 offers critical insights into the prevalence and characteristics of extended-spectrum beta-lactamase-producing Enterobacteriaceae (ESBL-E) in vegetables worldwide. This study focused specifically on studies reporting ESBL-E isolated from vegetable samples, as many articles were excluded for reporting only the prevalence of Enterobacteriaceae specifying without ESBL-producing strains. The inclusion criteria of this review emphasize the importance of understanding ESBL-E specifically, given its relevance to antimicrobial resistance and public health.

Geographically, most studies included in this review were conducted in Asian and European countries, likely reflecting the availability of research funding and the prioritization of antimicrobial resistance studies in these regions. Remarkably, studies have yet to be identified from Australia, which may suggest either a lack of research interest in this specific area or the under-recognition of ESBL-E in vegetables, with this being neglected as a significant issue within that continent.

The pooled prevalence estimates of ESBL-E in vegetables, based on 2762 positive isolates, were found to be 11.9%. This prevalence may be attributed to using wastewater and manure in agriculture, known sources of antibiotics and resistant bacteria in soil environments [81]. Comparatively, our findings reveal a lower prevalence than previously reported systematic studies by Pintor-Cora et al. [47] and Igbinosa et al. [34], who reported ESBL-producing Enterobacteriaceae in Spain (17.8%) and Nigeria (36.6%), respectively. Conversely, this prevalence is higher in estimates reported in studies from Japan and South Korea, with Usui et al. [8] and Song et al. [10] documenting prevalence rates of 7.7% and 0.83%, respectively. The discrepancy could be due to a difference in the timing of the study as well as differences in geographical characteristics, sampling categories and types, sample size, and identification methods.

The highest PPE and most significant number of studies on ESBL-E in vegetables were reported between 2022 and 2024. This could mean public awareness of the challenges associated with resistant bacteria in vegetables has recently grown. This could also be due to the availability of new and sophisticated detection methods in recent years. Additionally, experts are becoming increasingly aware of ESBL-E’s threat to vegetables. Possible reasons for this change could be the increased funding for research.

Among the ESBL genes analyzed, *bla*_CTX-M_ and *bla*_SHV_ were most frequently detected in Enterobacteriaceae isolated from vegetables, highlighting their widespread distribution in agricultural environments. Particularly, the *bla*_CTX-M_ and *bla*_SHV_ genes are widely distributed in vegetables. In studies employing PCR and whole-genome sequencing (WGS) molecular detection techniques, the CTX-M group (*bla*_CTX-M-1_, *bla*_CTX-M-14_, *bla*_CTX-M-15_, *bla*_CTX-M-55_, and *bla*_CTX-M-65_) was predominant. This high prevalence of CTX-M-type ESBL producers may be due to the global distribution of clones with epidemic and pandemic potential, such as the extraintestinal pathogenic *E. coli* (ExPEC) ST131, which is known for its ability to form extensive bacteria to produce a spectrum of β-lactamases, such as *bla*_CTX-M-15_ [13,82]. The *bla*_CTX-M_ enzymes are the most common type of ESBL because they are of an environmental origin [16,83]. International trends indicate that *bla*_CTX-M-15_ is the most prevalent ESBL gene on a global level [10]. We also report the presence of the *bla*_CTX-M-15_ gene in this systematic review. Correspondingly, *bla*_CTX-M-15_ was detected from Enterobacteriaceae isolated from environmental samples in Tunisia [84]. The *bla*_CTX-M-15_ gene is encoded horizontally by corresponding plasmids to several lineages [85,86]. Alternatively, the strains may be spreading through clonal expansion [86].

In addition to ESBL-producing genes, this review identified eight Enterobacteriaceae isolates harbouring the carbapenemase-encoding gene (*bla*_VIM_). This finding is consistent with worldwide patterns since the use of carbapenem to treat ESBL infections has been selectively pressuring the development of carbapenem resistance [87]. The World Health Organization has classified carbapenemase-producing Enterobacteriaceae as a critical priority due to their high resistance levels and the challenges they pose in clinical treatment [88]. This highlights the importance of continued surveillance and control efforts to mitigate the spread of carbapenem-resistant Enterobacteriaceae in agricultural and food settings [15].

ESBL-producing Enterobacteriaceae have plasmid-mediated enzymes that carry multi-resistance genes via integrons, transposons, and plasmids, and their multi-resistance can be explained by the ease with which they can be transferred to other bacteria through transformation, conjugation, or transduction [89]. Bacteria with multiple antibiotic resistance are now widely distributed in processed foods and have become a serious problem worldwide [21,90]. The overall pooled estimate of MDR-ESBL-producing Enterobacteriaceae in this study was 49.1%. The rate of MDR isolates in this study is lower than in some other vegetable studies [21,91]. The pattern of antibiotic resistance varies between regions and countries according to the level of antibiotic use, which is controlled and regulated by a particular country’s antibiotic policy [92,93].

Since soil bacteria, fungi, protists, small animals, and all plants contain antibiotic resistance determinants, soil can be considered a relatively large reservoir [94]. Soil can also serve as a reservoir for bacteria and antibiotic resistance genes because it contains microorganisms that produce antibiotics [95]. Animal manure that has been piled up may also contain antibiotics and their metabolites, which can leak into the soil, surface water, and groundwater [93]. Moreover, antibiotics may be accidentally released into the environment through farm runoff, irrigation with wastewater produced by agricultural operations, or soil fertilization with raw animal manure [96]. The aquatic system and sewage are the primary sources of antibiotic-resistance genes, which are produced by the use of antibiotics and the disposal of waste [52,97]. Additionally, fresh produce can become contaminated while being processed [52,98].

Contaminated vegetables are difficult to disinfect, especially if bacteria are established in plant tissue [23,99,100]. Furthermore, ESBL-E could spread easily, especially in areas with inadequate sanitation [101,102,103]. It is important to minimize contamination of fresh produce with pathogenic or antibiotic-resistant bacteria. This can be achieved by implementing good agricultural and manufacturing practices. In addition, studies should be conducted to investigate at which stages or critical points contamination of fresh produce with antibiotic-resistant bacteria is most likely to occur and to identify the sources of contamination to establish a better control system that ensures safer vegetables from farm to fork.

### Strengths and Limitations

This systematic review and meta-analysis quantitatively estimate the global burden of ESBL-E in vegetables. To our knowledge, this is the first attempt to investigate the global prevalence of ESBL-E in vegetables. The results of this study could be helpful to stakeholders in developing policies and implementing consumer awareness and food safety programs. Despite our efforts to document the global status of ESBL-E, it is essential to acknowledge certain limitations. Due to a lack of published studies, the pooled prevalence of some nations and continents needed to be calculated. Due to the limited number of studies, some genes associated with antibiotic resistance were also excluded from this meta-analysis. Meta-analyses that include fewer than ten studies or have a high degree of heterogeneity between studies may produce misleading results from these assessment tools. Evaluating the actual outcomes of statistically significant publication bias tests is challenging when there is a high degree of heterogeneity. Readers should exercise caution when interpreting pooled analyses and subgroups due to the high level of heterogeneity present in all analyses.

## 5. Conclusions

Based on the studies analyzed in this meta-analysis, the prevalence of ESBL-E in vegetables has increased over time, and wastewater and manure may be the main final repository of ESBLs. This study also noted the high frequency of CTX-M genes in Enterobacteriaceae isolated from vegetables. This is a worrying sign of the global spread of plasmids causing epidemic resistance. Data from other regions of the world should be used to implement strategic interventions to combat antimicrobial resistance, such as efficient infection prevention and control and prudent antibiotic use, to limit the spread of ESBL-E. The burden and risk posed by the carriage of ESBL genes cannot be accurately estimated due to the lack of molecular studies conducted worldwide. The role of vegetables in the spread of antibiotic-resistant bacteria should be further investigated to better address and control the problem of antibiotic resistance in the future.

## Figures and Tables

**Table 1:** Characteristics of the reviewed articles included in the meta-analysis.

Study ID	Years	Country	Total	No. of ESBL Positive	Prevalence (%)
Adhikari et al. [21]	2023	Nepal	72	37	51.4
Al-Kharousi et al. [22]	2019	Oman	105	27	25.7
Beshiru et al. [23]	2023	Nigeria	300	7	2.3
Bhutani et al. [24]	2015	USA	50	3	6.0
Blaak et al. [25]	2014	Netherlands	147	91	61.9
Boonyasiri et al. [26]	2014	Thailand	43	5	11.6
Cao et al. [27]	2023	China	576	4	0.7
Cha et al. [28]	2020	Korea	631	1	0.2
Chotinantakul et al. [29]	2022	Thailand	182	16	8.8
Colosi et al. [30]	2020	Romania	165	5	3.0
Freitag et al. [31]	2018	Germany	245	7	2.9
Geuther et al. [32]	2023	Rwanda	339	13	3.8
Harada et al. [33]	2017	Vietnam	84	9	10.7
Igbinosa et al. [34]	2023	Nigeria	78	30	38.5
Jung and Rubin [35]	2020	Canada	143	7	4.9
Kaesbohrer et al. [36]	2019	Germany	399	1	0.3
Kim et al. [37]	2015	South Korea	189	19	10.1
Kurittu et al. [38]	2021	Finland	60	3	5.0
Lv et al. [39]	2020	China	1340	233	17.4
Mesbah Zekar et al. [40]	2020	Algeria	310	12	3.9
Moon et al. [41]	2022	USA	88	10	11.4
Msimango et al. [42]	2023	South Africa	192	32	16.7
Njage and Buys [43]	2015	South Africa	10	10	100.0
Esch-inderbinen et al. [44]	2015	Switzerland	225	3	1.3
Ortega-Paredes et al. [45]	2018	Ecuador	27	3	11.1
Parker et al. [46]	2021	USA	364	23	6.3
Pintor-Cora et al. [47]	2021	Spain	117	31	26.5
Pintor-Cora et al. [48]	2023	Spain	145	24	16.6
Quarcoo et al. [49]	2022	Ghana	125	60	48.0
Rasheed et al. [50]	2014	India	30	2	6.7
Ratshilingano et al. [51]	2022	South Africa	136	7	5.1
Reuland et al. [52]	2014	Netherlands	119	7	5.9
Richter et al. [53]	2020	South Africa	545	61	11.2
Romyasamit et al. [54]	2021	Thailand	305	14	4.6
Saechue et al. [55]	2024	Thailand	93	6	6.5
Said et al. [56]	2015	Tunisia	45	9	20.0
Salmanov et al. [57]	2021	Ukraine	3326	1224	36.8
Sapkota et al. [58]	2019	Nepal	95	12	12.6
Song et al. [10]	2020	South Korea	1324	11	0.8
Usui et al. [8]	2019	Japan	157	10	6.4
de Godoy et al. [59]	2024	Brazil	23	13	56.5
Ye et al. [60]	2018	China	59	9	15.3
Zurfluh et al. [61]	2015	Dominican Republic, India, Thailand, and Vietnam	169	43	25.4
Achinike and Osakuade [62]	2023	Nigeria	30	7	23.3
Asfaw et al. [63]	2023	Ethiopia	176	23	13.1
Ashwath et al. [64]	2022	India	20	4	20.0
Díaz-Gavidia et al. [65]	2021	Chile	478	16	3.3
Ghimire et al. [66]	2020	Nepal	57	4	7.0
Gómez-Sanz et al. [67]	2023.	Spain	115	104	90.4
Huizinga et al. [68]	2018	Netherlands	131	25	19.1
Iseppi et al. [69]	2018	Italy	80	20	25.0
Kon et al. [70]	2023	Israel	301	20	6.6
Lee et al. [71]	2021	Malaysia	180	113	62.8
Montero, et al. [72]	2021	United States	117	11	9.4
Nayme et al. [73]	2017.	Morocco	144	4	2.8
Ojer-Usoz et al. [74]	2017	Spain	306	11	3.6
Shastry et al. [75]	2021	India	275	83	30.2
Sokmen and Özpinar [76]	2016	Turkey	108	69	63.9
Soré et al. [77]	2020	Burkina Faso	113	48	42.5
Toh et al. [78]	2018	Saudi Arabia	480	13	2.7
van Hoek et al. [79]	2015	Netherlands	63	21	33.3
Vázquez-López et al. [80]	2018	Mexico	12	12	100.0

**Table 2:** Subgroup analysis of ESBL-producing Enterobacteriaceae in vegetables.

Risk Factors	Number of Studies	Pooled Estimates			Measure of Heterogeneity			Publication Bias
		Sample Size	ESBL Positive	I^2^ (95%CI)	Q Value	I^2^	*Q*	Begg and Mazumdar Rank *p*-Value
**Overall**	62	16,363	2762	25.4% (9.5–16.2)	1968.339	96.901	<0.001	0.515
**Methods**								
DDST	22	4106	333	10.6% (6.6–16.6)	321.109	93.460	<0.001	0.516
Disk Diffusion	16	5021	1586	17.0% (11.7–24.1)	286.713	94.768	<0.001	0.021
CHROMagar ESBL	13	4676	589	12.6% (6.4–23.1)	538.316	97.771	<0.001	0.021
PCR	5	1033	147	21.1% (1.8–79.6)	215.571	98.1	<0.001	0.707
**Years**								
2014–2015	11	1090	213	14.2% (6.7–27.7)	219.392	94.986	<0.001	0.450
2016–2017	4	642	93	11.1% (1.4–53.1)	152.154	98.028	<0.001	0.496
2018–2019	12	2335	200	10.6% (6.3–17.3)	133.776	91.030	<0.001	0.360
2020–2021	17	9305	1838	9.8% (6.0–15.6)	861.136	98.026	<0.001	0.471
2022–2024	18	2991	417	15.9% (8.8–27.2)	462.581	96.325	<0.001	0.306
**Countries**								
China	3	1975	246	6.4% (1.3–25.9)	45.165	95.572	<0.001	0.117
India	3	325	89	19.5% (8.5–38.6)	6.596	69.676	<0.001	0.117
South Africa	4	883	110	13.7% (7.0–25.3)	21.938	86.325	<0.001	0.496
Netherlands	4	460	144	25.4% (8.6–55.0)	86.175	96.519	<0.001	0.174
Nepal	3	224	53	19.0% (4.3–54.9)	38.178	94.761	<0.001	0.601
Nigeria	3	408	44	14.3% (2.2–55.3)	53.278	96.246	<0.001	0.601
USA	4	619	47	7.9% (5.9–10.5)	3.277	8.463	<0.001	0.496
Spain	4	683	170	28.4% (5.7–72.3)	167.700	98.211	<0.001	0.500
**Continent**								
Africa	14	2543	323	14.3% (8.1–24.1)	287.412	95.477	<0.001	0.547
Asia	23	6727	727	10.7% (6.6–17.0)	706.086	96.743	<0.001	0.233
Europe	16	2951	372	11.0% (5.0–22.2)	475.649	96.636	<0.001	0.266
North America	5	762	54	7.4% (5.5–9.8)	4.708	15.031	<0.001	0.500
South America	4	3854	1256	19.4% (4.3–56.0)	130.790	97.706	<0.001	0.496

**Table 3:** Subgroup analysis of ESBL-resistant genes.

Risk Factors	No. of Studies	Pooled Estimates			Measure of Heterogeneity	Publication Bias
		Sample Size	ESBL Positive	I^2^ (95%CI)	I^2^	Begg and Mazumdar Rank *p*-Value
*bla*_Amp_C	15	3198	193	4.3% (2.5–7.1)	88.992	0.424
*bla*_CTX-M_	16	2768	100	6.5% (3.0–13.7)	90.057	0.787
*bla*_CTX-M-1_	7	610	34	7.9% (2.8–20.1)	85.112	0.880
*bla*_CTX-M-14_	7	383	44	18.6% (8.2–37.1)	84.543	0.452
*bla*_CTX-M-15_	19	274	91	31.0% (19.0–46.3)	72.496	0.344
*bla*_CTX-M-55_	5	322	15	10.8% (2.6–35.8)	85.084	0.327
*bla*_CTX-M-65_	3	65	6	9.9% (4.5–20.4)	0.000	0.601
*bla*_TEM_	16	2219	92	4.8% (2.4–9.3)	88.663	0.528
*bla*_SHV_	8	228	76	42.8% (26.9–60.3)	73.281	0.216
*bla*_SHV-2_	3	87	1816	21.7% (10.2–40.2)	62.333	0.601
*bla*_SHV-12_	6	121	16	15.1% (8.6–25.3)	24.182	0.850
*bla*_SHV-28_	4	111	13	14.6% (4.0–41.5)	79.127	0.174
*bla*_FONA-2_	5	297	16	12.1% (3.7–32.7)	77.946	0.327
*bla*_OXA-1_	5	97	18	25.7% (9.8–52.5)	72.641	0.500

## Data Availability

No new data were created or analyzed in this study. Data sharing is not applicable to this article.

## Supplementary Materials

The following supporting information can be downloaded at: https://www.mdpi.com/article/10.3390/foods13233961/s1, Supplementary Table S1. Checklist of items to include when reporting a systematic review or meta-analysis.
